# To bind or not to bind – how to down-regulate target genes by liganded thyroid hormone receptor?

**DOI:** 10.1186/1756-6614-1-4

**Published:** 2008-10-11

**Authors:** Joachim M Weitzel

**Affiliations:** 1 Institute of Experimental Endocrinology, Charité University Medicine Berlin, 13353 Berlin, Germany; 2 Department of Reproductive Biology, FBN Dummerstorf, 18196 Dummerstorf, Germany

## Abstract

The terrain is well explored regarding genes whose gene expression is up-regulated upon binding of thyroid hormone (TH) to its nuclear receptor. This regulation mechanism has been intensively studied and is well understood. In contrast, a lot of white spots remain on the map when it comes to target genes whose expression is down-regulated upon binding of TH to the thyroid hormone receptor (TR). Since no consistent mechanism has been proposed to explain ligand-dependent down-regulation of target gene transcription several working hypotheses favour different molecular mechanisms. Some working theories suggest a direct binding of TR to regulatory elements of target genes. Others favour models that are independent of a direct DNA binding event. However recent data suggested that a direct binding of TR to DNA is dispensable for TH-dependent negative gene transcription.

## Introduction

### Regulation of gene expression in response to TH

#### a) Up-regulation by TH – the easy story

Thyroid hormone regulates gene expression in a positive and negative manner. The mechanism of positively TH-regulated gene transcription has been intensively studied and is well understood. The thyroid hormone receptor (TR) binds to thyroid hormone response elements (TREs) which are located within regulatory elements of TH target genes. If the ligand (i.e. triiodothyronine; T3) is present, liganded TR recruits a huge coactivator complex. This coactivator complex possesses or recruits several enzymatic activities which modify the chromatin of target genes generating an open structure allowing for transcription. If the ligand is absent the un-liganded TR undergoes a conformational change which releases the coactivator complex and recruits a corepressor complex. Again this corepressor complex integrates several enzymatic activities modifying chromatin toward a closed and transcriptional silent state. These processes are well documented and discussed in comprehensive and excellent review articles elsewhere; see e.g. [1-3].

#### b) Down-regulation by TH – the complicate story

**Figure 1:**
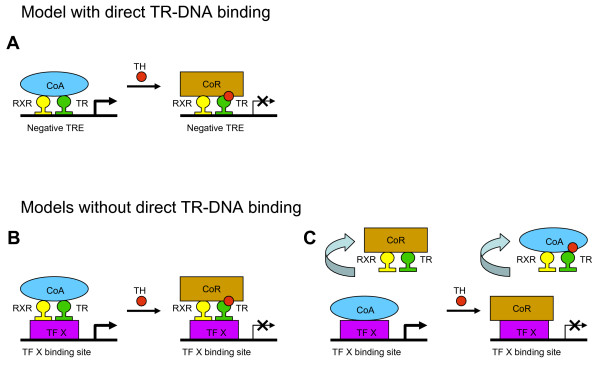
**Alternative models for negative gene transcription by thyroid hormone**. A) Thyroid hormone receptor (TR) binds together with its dimerization partner RXR to a negative thyroid hormone response element (negative TRE). The un-liganded TR recruits a coactivator complex (CoA) leading to gene activation. If thyroid hormone (TH) binds to TR the liganded TR recruits a corepressor complex (CoR) leading to silencing of gene expression. B) TR does not bind directly to DNA but rather via unknown transcription factors (TF X). The functional readout is as described for model A. C) TR does not bind to DNA at all but sequesters CoR away from DNA-bound transcription factors (TF X) in the absence of TH. In the presence of TH, liganded TR sequesters CoA away from DNA-bound transcription factors. For further explanation, see text.

Besides positively TH-regulated target genes there are clearly those genes whose gene expression is negatively regulated upon administration of TH. The probably best known example is the down-regulation of thyrotropin (TSH) in the pituitary as part of the negative endocrine hypothalamus-pituitary-thyroid feedback loop. However also in other tissues (e.g. the liver) negative gene regulation is a well known regulation pattern in microarray analysis [4-8]. The portion of negatively regulated genes varies greatly between 20 to >50% in different studies depending on the experimental design (e.g. the procedure to induce hypothyroidism in the animals or the duration, concentration and nature of TH treatment). From a mechanistically point of view, it should be noted that the down-regulation phenomenon is preserved in transient transfection experiments in cell culture, thus other in vivo relevant aspects of TH metabolism such as local TH availability controlled by membrane transporters or local activation/inactivation by deiodinases could be ignored in this experimental setting. Since positive and negative regulation takes place within the same cell system the divergent regulation mechanisms can be ascribed to the nature of different DNA response elements. The question remains how this negative regulation might occur. In principle there are three major working hypotheses trying to explain negative TH-mediated gene transcription. One of them (model A) suggests a direct binding of TR to DNA, whereas models B and C favour mechanisms without a direct TR-DNA binding (Figure 1).

(A) TR binds to specific negative thyroid hormone response elements (negative TREs). Due to the specific composition of these DNA response elements the TR undergoes specific conformational changes leading to a recruitment of a coactivator complex in the absence of TH. In contrast, binding of the ligand recruits a corepressor complex to the liganded TR. Of note, the functional readout is diametrically opposed compared to the readout for positively regulated genes (see above).

(B) A second model suggests that TR does not bind to DNA directly but rather to another transcription factor via protein-protein interactions. Again, the un-liganded TR (bound to DNA via transcription factor X) recruits a coactivator complex whereas the liganded TR recruits a corepressor complex.

(C) Finally, a third model suggests that a soluble, not DNA-bound TR sequesters cofactors away from other DNA-bound transcription factor-cofactor complexes. Sequestering of corepressor components from DNA-bound transcription factors finally leads to an activation of gene expression whereas sequestering of coactivators leads to a repression of gene transcription.

### Pros and cons of the three suggested models

Several pros and cons exist for all of the three models and no uniform model is in sight which takes in account all aspects of negative gene regulation.

#### Model A

Several reports described negative TREs (Figure 1, model A) on the basis of classical reporter gene assays in cell culture. DNA sequences close to the transcriptional start site (the so-called z-boxes) have been suggested to serve as negative TREs [9-17]. However; consecutive 5' deletion of promoter sequences to the transcriptional start site or introducing of point mutations at this site dampened the reporter gene activity close to background activities. Thus a potential down-regulation effect (which is probably still present) might simply be lost in the background noise of the remaining promoter activity. Furthermore, introducing of a z-box into a heterologous promoter context could not preserve a down-regulation effect (an assay which is routinely used for positive TREs). Moreover, a direct binding of TR could not be detected in mammalian one-hybrid cell experiments using a chimeric fusion protein of TR with the viral activator domain VP16 which activates gene transcription TH-independently [12,18,19]. Finally, a high-affinity binding of TR to a negative TRE has not been identified in biochemical assays using in vitro translated or bacterially expressed TR. Compared to classical positive TREs the binding affinities of negative TREs are at least two orders of magnitudes lower [9,12,14,15,17].

One major support for the model of negative TREs came from experiments by Frederic Wondisford and colleagues. They investigated the double mutant E125G; G126S within TRβ (so-called GS mutant) which conserved the overall zinc finger structure of the DNA binding domain (DBD) but altered the DNA binding specificity [20]. The GS mutant did not bind to a classical DR+4 TRE but rather to a mixed thyroid hormone/glucocorticoid hormone response element. However, the GS mutant still activates some major TH target genes which contain a mixed thyroid/glucocorticoid hormone response element (DR+4+2 element) [21,22] (unpublished data). This might be connected to the phenotype of GS mutant knock-in mice which show some but not all phenotypical alterations compared to classical TRβ knock-out mice [23]. Recent data from our laboratory indicated that targeting of full-length TR to DNA via a heterologous Gal4-DBD leads to an activation of gene expression in response to TH [19]. This finding is consistent with data using a similar experimental system in transgenic mice [24].

#### Model B

If TR did not bind directly to DNA it might bind via protein-protein interactions to a transcription factor as a bridging factor in order to target TR to specific regulatory elements (Figure 1, model B). Several recent publications support this idea opening the avenue for cell type specific regulation. One example might be the interaction of TR with the pituitary-specific transcription factors GATA2 and Pit1, which are important regulators of TSH transcription [25,26]. TR has been shown to physically interact with GATA2 and this is ensured via the DBD of TR [26]. Another interesting connection might be the antagonism of the cAMP pathway with the TH pathway which is accomplished by physical interaction between CREB and TR which – again – is mediated via the DBD of TR [13,27]. In addition TR-DBD might have several non-genomic effects, e.g. antagonising β-catenin activation via increased proteasomal degradation [28]. Many other examples of TR-DBD interactions with other transcription factors or chromatin components have been described and – interestingly – some of these protein-protein contacts appear to be ligand-dependent. Only in the presence of thyroid hormone TR and HDAC2 are recruited to the TSHβ promoter and a biochemical fine mapping identified the TR-DBD as binding domain for HDAC [9]. A ligand-dependent recruitment of HDAC would help to explain silencing of target gene transcription in response to TH. Other examples are the MED1/TRAP220 nucleosome remodelling complex and the insulator protein CTCF. The MED1/TRAP220 complex re-positioned nucleosomes on the Crabp1 gene in a TH-dependent manner which leads to an altered access of the basal transcription machinery and consequently to altered transcription rates. However, the role of TR within this process is not clear and both gene activation and gene repression in response to TH has been reported [29,30]. The insulator protein CTCF dampens gene transcription in an enhancer blocking assay only if liganded-TR is bound to an adjacent TRE. If the ligand is absent the enhancer activity could not be blocked by CTCF [31]. With other words, gene transcription rates are high in the absence of TH and low in the presence of TH – the typical readout of a negatively regulated gene. All these data suggested a role for TR and TH in negative gene transcription; however, it remains to be elucidated whether the DBD of TR is responsible for direct DNA binding and/or protein-protein interaction. Unfortunately, currently widely used chromatin immunoprecipitation assays could not distinguish between those factors which are directly bound to DNA and those factors which are indirectly bound to DNA as part of the chromatin complex. However, a modification of local chromatin structure has to be kept in mind to understand these processes.

#### Model C

Following an alternative hypothesis TR might not bind to DNA at all (neither directly (model A) nor indirectly (model B)), but rather sequesters cofactors from DNA-bound transcription factor-cofactor complexes (Figure 1, model C). Such a scenario has been demonstrated for corepressor NCoR and coactivator SRC-1 which are directed to DNA via a heterologous Gal4-DBD. In this experimental setting TR represses gene transcription of a reporter gene in response to TH without directly binding to DNA [18,19]. Soluble, non-DNA-bound TR might compete for limiting amount of cofactors, e.g. for SRC-1, a major coactivator for TR. In line with this argumentation SRC-1 knock-out mice show features of thyroid hormone resistance including several defects to properly regulate gene expression in response to TH [32]. Furthermore, TRβ ^E457A ^knock-in mice harbouring a defective coactivator binding site demonstrated alterations in positively and – paradoxically – negatively regulated TH target genes [33]. On the other hand the corepressor NCoR appears to be also essential for down-regulation since knock-down prevents negative gene regulation whereas re-introducing of NCoR reconstitutes this activity [14,34]. It remains unclear whether TR is part of the local chromatin complex or whether or not TR is directly or indirectly bound to DNA. If TR did not bind directly to target DNA (model B) the problem to specifically regulate only a subset of target genes arises. One could speculate that a yet to defined subset or combination of transcription factors or cofactors might be critical for regulating these target genes. However, the specificity problem emerges even more if we postulate a non-DNA bound TR (model C).

## Further perspectives

Up to now no convincing data have been presented doubtlessly supporting one particular model by discarding hitherto alternative models. Taking together recent data a direct binding of TR to negative TREs (model A) appears to be unlikely due to the lack of high-affinity binding sites for TR. Rather TR is involved in modulating the transcriptional activity of other transcription factors either directly (model B) or indirectly (model C). Clearly the DBD of TR participates in these processes independent of its function to bind DNA. Furthermore, cell-type and/or context-specific post-translational modifications might additionally contribute. Identification of TR binding sites by chromatin immunoprecipitation and correlation with adjacent DNA sequences (as successfully performed for the estrogen receptor [35]) are eagerly awaited to substantiate models for negative gene regulation by thyroid hormone.

## Competing interests

The author declares that they have no competing interests.
